# Value of Fused ^**18**^F-Choline-PET/MRI to Evaluate Prostate Cancer Relapse in Patients Showing Biochemical Recurrence after EBRT: Preliminary Results

**DOI:** 10.1155/2014/103718

**Published:** 2014-04-30

**Authors:** Arnoldo Piccardo, Francesco Paparo, Riccardo Picazzo, Mehrdad Naseri, Paolo Ricci, Andrea Marziano, Lorenzo Bacigalupo, Ennio Biscaldi, Gian Andrea Rollandi, Filippo Grillo-Ruggieri, Mohsen Farsad

**Affiliations:** ^1^Nuclear Medicine Department, E.O. Galliera Hospital, Mura delle Cappuccine 14, 16128 Genoa, Italy; ^2^Radiology Department, E.O. Galliera Hospital, Mura delle Cappuccine 14, 16128 Genoa, Italy; ^3^Radiotherapy Department, E.O. Galliera Hospital, Mura delle Cappuccine 14, 16128 Genoa, Italy; ^4^Nuclear Medicine Department, Azienda Sanitaria dell'Alto Adige, Via Lorenz Böhler 5, 39100 Bolzano, Italy

## Abstract

*Purpose*. We compared the accuracy of ^18^F-Choline-PET/MRI with that of multiparametric MRI (mMRI), ^18^F-Choline-PET/CT, ^18^F-Fluoride-PET/CT, and contrast-enhanced CT (CeCT) in detecting relapse in patients with suspected relapse of prostate cancer (PC) after external beam radiotherapy (EBRT). We assessed the association between standard uptake value (SUV) and apparent diffusion coefficient (ADC). * Methods*. We evaluated 21 patients with biochemical relapse after EBRT. Patients underwent ^18^F-Choline-PET/contrast-enhanced (Ce)CT, ^18^F-Fluoride-PET/CT, and mMRI. Imaging coregistration of PET and mMRI was performed. * Results*. ^18^F-Choline-PET/MRI was positive in 18/21 patients, with a detection rate (DR) of 86%. DRs of ^18^F-Choline-PET/CT, CeCT, and mMRI were 76%, 43%, and 81%, respectively. In terms of DR the only significant difference was between ^18^F-Choline-PET/MRI and CeCT. On lesion-based analysis, the accuracy of ^18^F-Choline-PET/MRI, ^18^F-Choline-PET/CT, CeCT, and mMRI was 99%, 95%, 70%, and 85%, respectively. Accuracy, sensitivity, and NPV of ^18^F-Choline-PET/MRI were significantly higher than those of both mMRI and CeCT. On whole-body assessment of bone metastases, the sensitivity of ^18^F-Choline-PET/CT and ^18^F-Fluoride-PET/CT was significantly higher than that of CeCT. Regarding local and lymph node relapse, we found a significant inverse correlation between ADC and SUV-max. * Conclusion*. ^18^F-Choline-PET/MRI is a promising technique in detecting PC relapse.

## 1. Introduction

In patients affected by prostate cancer (PC) and treated with external beam radiation therapy (EBRT) relapse may occur in about 50% of cases within 5 years after treatment [1] and the recurrence rate of PC in these patients is reasonably higher than in those treated with surgery. Increasing levels of PSA after EBRT are often related to PC recurrence, especially when PSA rises above 2 ng/mL [2] and PSA doubling time is <6 months [3].

Several imaging techniques have been used to detect sites of PC recurrence, such as bone scintigraphy (BS), contrast-enhanced computed tomography (CeCT), and ultrasound (US). CeCT and US do not provide high diagnostic accuracy in detecting relapse [4, 5]. BS, which has low specificity, often requires further adequate investigation. In this field, ^18^F-Fluoride-PET/CT may be helpful, especially in high risk PC patients, as its high sensitivity and specificity in detecting bone lesions overcome the intrinsic limitations of BS [6]. At present, the most promising tools are multiparametric MRI (mMRI) and PET/CT with radio-labelled choline derivatives. MRI can accurately detect residual prostate cancer and locoregional recurrence after EBRT [7]. Choline-PET/CT, being a whole-body imaging technique, may locate lymph nodes and bone metastases in the majority of patients treated with EBRT [8–10]. The simultaneous acquisition of PET and mMRI with new PET/MRI scanners may be helpful, especially in doubtful cases [11], but this integrated imaging modality is currently not easily available. To date, however, little can be said about the accuracy of combined ^18^F-Choline-PET/MRI in PC [12] and few studies have compared the accuracy of mMRI and ^18^F-Choline-PET/CT in patients affected by recurrent PC [13].

The aim of this study was firstly to assess the diagnostic performance of fused ^18^F-Choline-PET/MRI in patients with suspected relapse of PC after EBRT. We also compared the accuracy of ^18^F-Choline-PET/MRI with that of mMRI, ^18^F-Choline-PET/CT, ^18^F-Fluoride-PET/CT, and CeCT in detecting local recurrence, lymph node, and bone metastases. We finally assessed the association between the standard uptake value (SUV-max) of PET and apparent diffusion coefficient (ADC) value of local recurrence and lymph node metastases.

## 2. Materials and Methods

### 2.1. Patient Population

We prospectively evaluated 21 PC patients who showed biochemical relapse after first-line treatment with EBRT. Sixteen patients were treated only with EBRT and the others were treated with EBRT and androgen deprivation therapy (ADT). We included in our study only patients with Gleason score ≥7, PSA ≥ 2 ng/mL, and PSA doubling time (DT) ≤6 months. All patients underwent integrated ^18^F-Choline-PET/contrast-enhanced (Ce)CT, ^18^F-Fluoride-PET/CT, and mMRI within ten days. The main characteristics of the patients are summarized in Table 1. The Local Ethics Committee approved the study. All patients had been treated exclusively with EBRT as primary treatment. Patients had received a dose range of 74–81 Gy to the prostate gland and 72–74 Gy to the prostatic fossa. Therapy was performed in accordance with EAU guidelines [14]. All patients at the time of EBRT were classified as N0.

### 2.2. ^18^F-Choline-PET/CeCT Protocol

^18^F-Choline-PET/CT was performed in the fasting state (at least 6 h). An ^18^F-Choline activity of 3 MBq/kg (IASOCholine IASON Labormedizin Gesmbh & Co. Kg, Linz, Austria) was administered intravenously; data were acquired 10′ after the injection by means of a dedicated PET/CT system (Discovery ST; General Electric Healthcare Technologies, Milwaukee, WI). Low dose CT was acquired for both attenuation correction and topographic localization. The CT parameters used for acquisition were 140 kV, 80 mA, and 0.5 s per rotation and pitch 6 : 1, with a slice thickness of 3.25 mm equal to that of PET. PET was acquired in 3D mode from the upper neck to the upper thighs, by means of sequential fields of view, each covering 12 cm (matrix of 256 × 256), over an acquisition time of 3 min. Finally, a whole-body CeCT (120 kVp, *z*-axis tube current modulation with minimum/maximum mA: 200/600) was acquired during the portal venous phase, 50 s after reaching the threshold of 100 UH in the mid thoracic aorta using a bolus tracking technique. Iopromide 370 mg I/mL (Ultravist, Bayer HealthCare Pharmaceuticals Inc., Montville, NJ) was used as intravenous iodinated contrast medium in all patients, and it was administered at a fixed flow rate of 3 mL/s to guarantee a constant iodine delivery rate of 1.2 g I/s. ^18^F-Choline-PET/CT, CeCT, and ^18^F-Choline-PET/CeCT studies were visualized on Xeleris Workstation version 2.1753 (General Electric, Milwaukee, WI, USA) which allowed PET/CT and PET/CeCT fusion and CeCT multiplanar reconstructions.

### 2.3. ^18^F-Fluoride-PET/CT Protocol

PET acquisition started 60 min after intravenous injection of 370 MBq ^18^F-labelled Na F (IASOflu-IASON GmbHGraz, Austria) and included 10–12 bed positions. All PET scans were acquired in 3D mode (3 min emissions per bed position) and were reconstructed by using an iterative reconstruction algorithm. CT acquisition data were optimized to obtain diagnostic CT images also with small image reconstruction thickness using the following data: 120 kV, 120–400 mA with an automatic system of dose optimisation (Auto-mA, General Electric Medical Systems), tube rotation time 0.5 s, 16 mm × 1.25 mm detector configuration, and table feed 13.75 mm per rotation. Overall, we acquired only one CT scan, obtaining two image reconstruction thicknesses 3.75 mm (for attenuation correction and anatomic localization) and 1.25 mm (for multiplanar reconstruction). No iodinated contrast medium was injected. CT acquisition was performed from the skull vertex to the distal femur, with the same field of view used for ^18^F-Fluoride-PET.

### 2.4. Multiparametric MRI

Multiparametric MRI (mMRI) was performed with a 1.5T MRI scanner (Signa HDxt, GE Healthcare, Milwaukee, WI) equipped with 8-channel pelvic phased-array coil, according to a standardized protocol. FSE T2-weighted (T2w) sequences (slice thickness 4 mm, interslice gap 0.4 mm, in plane resolution 0.6 × 0.6 mm) with a large field of view were first oriented in the three orthogonal planes (sagittal, axial, and coronal), without fat saturation, enabling identification of the prostate gland and pelvic lymph node stations. High-resolution oblique axial and coronal scans (slice thickness 3–3.5 mm, interslice gap 0.3 mm) were further oriented perpendicular and parallel to the rectoprostatic plane, in order to avoid misinterpretation due to partial volume effects. Diffusion-weighted imaging (DWI) sequence (b 1000 s/m^2^, TR 3400, TE 76.3, bandwidth 250 kHz, FOV 40 × 40, slice thickness 4.0 mm, interslice gap 0.4 mm, matrix 128 × 128, number of excitations 6, and in plane resolution 1.5 × 1.5 mm) was acquired in the axial plane, using same slice locations of the first FSE T2w sequence. Dynamic contrast-enhanced MRI (DCE-MRI) was acquired during intravenous injection of the paramagnetic contrast medium with a flow rate of 3 mL/s. A 3-dimensional spoiled gradient echo fat saturated T1-weighted (LAVA) pulse sequence was repeated in the axial plane 27 times with a temporal resolution of 12 s, during the injection of a single dose of contrast agent. An axial STIR sequence (TR, 5000 ms; TE, 80.1 ms; inversion time, 150 ms; thickness, 5 mm; interslice gap 0.5 mm; matrix, 256 × 256) was performed to detect focal bone lesions and for comparing mMRI with PET techniques (i.e., ^18^F-Choline-PET/CT and ^18^F-NaF PET/CT) in the detection of bone recurrence. Antiperistaltic drugs were given for minimizing motion artifacts due to intestinal peristalsis at the beginning of each mMRI examination.

### 2.5. Fused ^18^F-Choline-PET/MRI

Multimodal imaging coregistration, fusion, and synchronized navigation were performed using a novel dedicated software, developed for research purposes (Quanta Prostate, Camelot Biomedical Systems s.r.l., Genoa, Italy). This software was developed for aiding multiparametric MRI (mMRI) interpretation and reporting, as well as multimodal MRI/PET coregistration. Quanta Prostate is able to simultaneously display different MRI datasets, allowing calculation of color-coded ADC maps from DWI sequences and wash-in/wash-out rate maps from time-intensity curves of DCE-MRI. Color-coded ADC and perfusion maps can be overlapped on T2w images and examined with different levels of transparency. Using Quanta Prostate, a deformable registration technique (employing nonlinear transformation and spatially varying deformable models) is applied for MRI/PET coregistration, in order to compensate for changes in patient positioning and local deformations between different imaging datasets (e.g., due to varying degrees of filling of the urinary bladder). Once MRI/PET coregistration is performed, the operator can perform quantitative measurements (ADC and SUV-max values) by drawing a ROI on the currently selected ADC or SUV map.

## 3. Image Interpretation

### 3.1. ^18^F-Choline-PET/CT and ^18^F-Fluoride-PET/CT

^18^F-Choline-PET/CT and ^18^F-Fluoride-PET/CT studies were interpreted visually and semiquantitatively using the maximum standardized uptake value (SUV-max), on a patient-by-patient and lesion-by-lesion basis using the above mentioned dedicated software to review fused PET/CT images. However, no SUV-max cut-off value have been introduced and SUV was calculated for each lesion just to support visual interpretation.

On ^18^F-Choline-PET/CT, any focal and nonphysiological uptake corresponding to prostate parenchyma, abdominal and pelvic lymph nodes, and bone was considered as pathological.

On ^18^F-Fluoride-PET/CT bone lesions were considered to be malignant if a high and focal ^18^F-Fluoride uptake was detected without any morphological changes on CT or a high and focal ^18^F-Fluoride uptake was associated to typical morphological changes on CT.

### 3.2. Multiparametric MRI

All mMRI were reviewed on a dedicated workstation on (Advantage Workstation 4.6, General Electric Medical Systems), using a tool for mMRI visualization and reporting. A definitive diagnosis of cancer recurrence (i.e., local, lymph nodal, and skeletal recurrence) was formulated after examining each MRI examination, reporting the number of lymph nodal and skeletal lesions. Findings indicative of local recurrence were identified according to ESUR prostate MR guidelines, 2012, considering that radiation-induced fibrosis and shrinkage of the gland may often hinder lesion detection [15]. DWI sequence was examined by generating an ADC map, searching for intraglandular nodular foci characterized by restriction of water diffusion relative to the surrounding prostate parenchyma. ADC value of the glandular area affected by recurrent disease was registered and compared with the corresponding SUV-max value of ^18^F-Choline-PET/CT. On DCE-MRI sequences, prostate cancer recurrence was recognized as an early enhancing area in contrast to the surrounding glandular tissue. A quantitative wash-in perfusion map was generated for aiding the detection of hypervascularized foci within the prostate gland. The following morphological and size criteria were used to define a metastatic lymph node: short axis diameter >10 mm for an oval lymph node and diameter >8 mm for a round lymph node. The ADC value of the most PET-positive lymph node of each lymph nodal station involved by recurrent disease was registered and compared to the SUV-max value of ^18^F-Choline-PET/CT. Axial STIR sequence was employed to search bone metastases that appear as hyperintense focal lesions within the spongy or cortical bone compartments.

### 3.3. Contrast-Enhanced CT

On CT scans, the separation between the prostate and the levator ani muscle is poorly defined, and intraprostatic anatomy is not well demonstrated. Only the following morphologic criteria were applied for defining local recurrence: prostate enlargement with focal capsule bulging; nodular areas of inhomogeneous contrast enhancement within the peripheral gland; sign of extracapsular extension. Bone metastases were considered to have an osteosclerotic or mixed (i.e., osteolytic and osteosclerotic) appearance.

### 3.4. Multimodal Fusion Imaging

On ^18^F-Choline-PET/MRI, any focus of nonphysiological uptake corresponding to any suspected mMRI finding in prostate gland was considered suggestive for local recurrence. Any focal uptake corresponding to mMRI detectable abdominal and pelvic lymph nodes was considered as lymph node metastasis. Any focal ^18^F-Choline bone uptake corresponding or not to a pathological finding on mMRI was considered as bone metastasis.

### 3.5. Standard of Reference

CeCT and mMRI at 12 months served as the standard of reference for the final discrimination between true positive, true negative, false positive, and false negative results. Further available follow-up information was provided by response to salvage therapy, laboratory tests/tumour markers, US-guided transrectal prostate biopsy, and other imaging studies (X-ray studies and bone scans). Patients and lesions were considered true negative if follow-up cross-sectional imaging studies and transrectal ultrasound were both negative and the patients had stable PSA values. A median clinical and imaging follow-up time of 14 months (range 12–18) was available for each patient.

Histopathologic confirmation was available in 3 out of 5 patients showing only local recurrence and in one out of six patients affected by lymph node metastases.

### 3.6. Statistical Analysis

No specific sample size calculation was performed, given the pilot nature of the study. Categorical data were summarised as number (percentage) of subjects/lesions; continuous data were summarised as mean, standard deviation, median and range The normal distribution of datasets of different variables was assessed by means of the D'Agostino-Pearson test. When datasets did not follow the normal distribution, nonparametric tests were used instead of parametric ones. The degree of correlation between ADC and -max values for local and lymph node recurrence was assessed by the Spearman rank test. The diagnostic performance of different diagnostic modalities (i.e., mMRI, CeCT, ^18^F-Choline-PET/CT, and ^18^F-NaF PET/CT) for determining the presence of recurrent disease was calculated using 2 × 2 tables. Sensitivity, specificity, positive and negative predictive values, and overall diagnostic accuracy were calculated. The diagnostic performances of different modalities were compared by means of the Fisher exact test for proportions with level of statistical significance set at 0.05.

## 4. Results

### 4.1. Patient-Based Analysis

Fused ^18^F-Choline-PET/MRI was positive in 18 of 21 patients, with an overall detection rate (DR) of 86%. Local relapse was detected in 6 patients, one of whom was also affected by bone metastases. Lymph node metastases were detected in 6 patients, two of whom also had bone involvement. Six patients showed only bone metastases. The DR of ^18^F-Choline-PET/CT, CeCT, and mMRI was 76% (16/21 patients), 43% (9/21 patients), and 81% (17/21 patients), respectively. The difference between the DR of multimodal fusion imaging ^18^F-Choline-PET/MRI and that of CeCT was statistically significant (*P* = 0.016), while, comparing mMRI and ^18^F-Choline-PET/CT with CeCT, it only tended to be significant (*P* = 0.093 for both modalities). No significant difference was found between ^18^F-Choline-PET/MRI and ^18^F-Choline-PET/CT, nor between ^18^F-Choline-PET/MRI and mMRI (*P* = 0.67 for both comparisons).  Table 2 shows the DRs recorded when the different sites of PC recurrence were considered separately. Considering all skeletal metastases, including those beyond the field-of-view (FOV) of mMRI and multimodal fusion imaging ^18^F-Choline-PET/MRI, ^18^F-Fluoride-PET/CT provided the highest DR (detecting all 9 patients affected by bone metastases), which was found to be significantly different from that of CeCT (*P* = 0.034).

### 4.2. Lesion-Based Analysis

Overall, 133 lesions were detected in our analysis, 102 of which were found in the pelvis and lower abdominal quadrants. Of these, 79 were malignant (including local recurrences and lymph node and skeletal metastases) and 54 were benign according to the standard of reference. Specifically, we detected 6 local recurrences, 40 pelvic lymph node metastases, and 33 bone metastases. Sixty of these 79 lesions were found in the field-of-view (FOV) of mMRI encompassing pelvis and lower abdominal quadrants. Considering only the FOV of mMRI, the overall accuracy of fused ^18^F-Choline-PET/MRI, ^18^F-Choline-PET/CT, CeCT, and mMRI was 99%, 95%, 70%, and 85%, respectively. Sensitivity, specificity, PPV, and NPV of different diagnostic modalities were as follows: 100%, 98%, 98%, and 100% for fused ^18^F-Choline-PET/MRI; 95%, 98%, 98%, and 93% for ^18^F-Choline-PET/CT; 50%, 100%, 100%, and 58% for CeCT; 75%, 95%, 96%, and 75% for mMRI.

**Figure 1:**
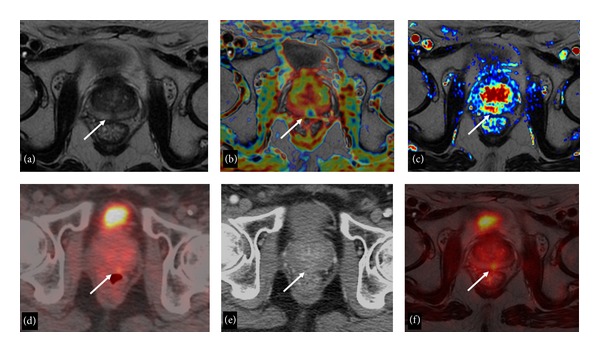
72-year-old man with biopsy-proven local PC recurrence after EBRT. T2-weighted axial image (a) demonstrates a hypointense focal area close to the midline (arrow). The ADC map (b) demonstrates that the nodular area (arrow) has significantly lower ADC values than the surrounding parenchyma. The wash-in perfusion map (c) shows a high wash-in rate (arrow). On the ^18^F-Choline-PET/CT axial image (d) a doubtful PET-positive focus (arrow) is appreciable, while the lesion is not detectable on CeCT axial image (e). Fused ^18^F-Choline-PET/MRI image (f) demonstrates precise correspondence between PET-positive focus and MRI finding (arrow).

**Figure 2:**
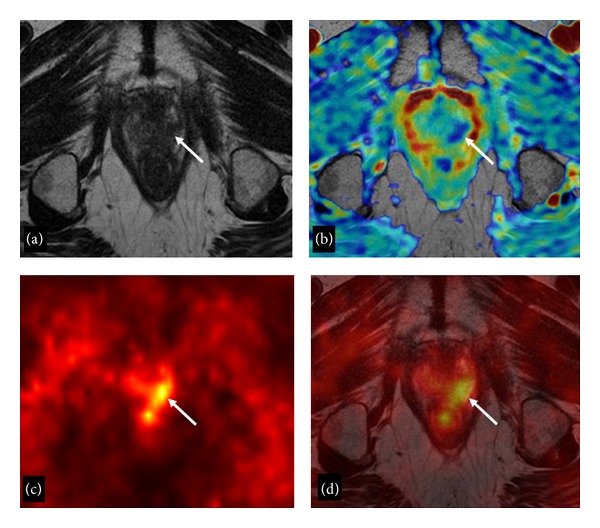
75-year-old man with biopsy-proven local PC recurrence after EBRT. T2-weighted axial image (a) demonstrates a hypointense area (arrow) in the posterior left lateral aspect of the prostate apex. The ADC map (b) demonstrates that the hypointense nodular area has lower ADC values than the surrounding parenchyma (arrow). On ^18^F-Choline-PET axial scan (c) a well-defined PET-positive focus is appreciable (arrow). Fused ^18^F-Choline-PET/MRI image (d) shows exact correspondence between PET-positive focus and MRI finding (arrow).

**Figure 3:**
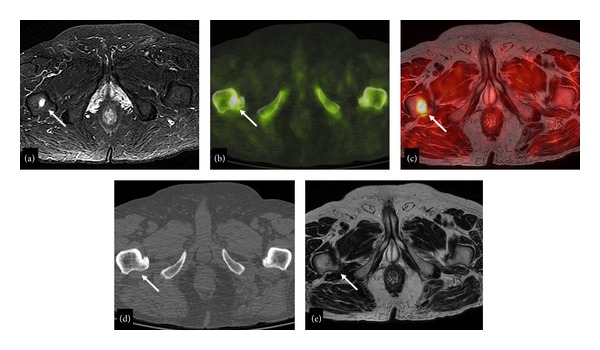
78-year-old man with a bone metastasis in the right proximal femur. The bone lesion (arrow) is well detectable on STIR (a), ^18^F-Fluoride-PET/CT (b), and fused ^18^F-Choline-PET/MRI axial images, while it is not visible on T2-weighted (d) and CeCT (e) axial images.

**Figure 4:**
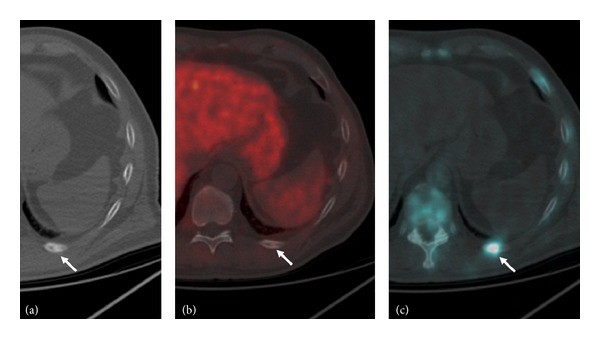
82-year-old man with a bone metastasis in the posterior arch of the left 10th rib. The lesion (arrow) is not definitely appreciable on the CeCT axial image (a) and is not detectable on the ^18^F-Choline-PET axial scan (b). On the ^18^F-Fluoride-PET/CT axial image (c) the skeletal metastasis is well detectable.

**Figure 5:**
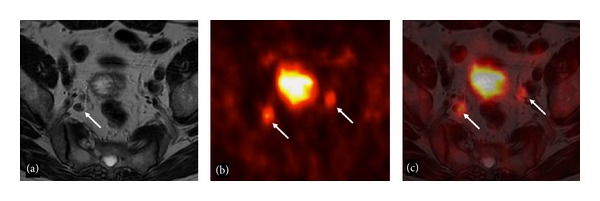
79-year-old man with bilateral hypogastric lymph node metastases. T2-weighted axial image (a) shows a right hypogastric lymphadenopathy (arrow). ^18^F-Choline-PET axial scan (b) demonstrates two areas of focal tracer uptake (arrows). Fused ^18^F-Choline-PET/MRI axial image (c) demonstrates that the left focus of tracer uptake corresponded to a very tiny hypogastric lymphadenopathy (arrows).

The accuracy, sensitivity, and NPV of fused ^18^F-Choline-PET/MRI were significantly higher than those of both mMRI (*P* < 0.05) and CeCT (*P* < 0.05). No differences in terms of specificity (*P* > 0.05) and PPV (*P* > 0.05) were observed between the different diagnostic modalities. A lesion-based analysis to determine the sensitivity of each modality in detecting recurrent lesions according to different anatomical locations (i.e., local recurrence, lymph nodes, and bone metastases) was also performed; these results are summarized in Table 3. ^18^F-Choline-PET/MRI was more useful in detecting local relapse, identifying more local recurrences (6/6, 100%), than ^18^F-Choline-PET/CT (4/6, 67%). Two cases of local recurrence are illustrated in Figures 1 and 2. However, the statistical difference between these two modalities was not significant, owing to the low number of local recurrences (*P* = 0.45). Fused ^18^F-Choline-PET/MRI showed significantly higher sensitivity in locating lymph node metastases than mMRI (*P* = 0.0002) and CeCT (*P* < 0.0001). One case is illustrated in Figure 3. On the other hand, multimodality fusion ^18^F-Choline-PET/MRI and ^18^F-Choline-PET/CT detected the same number of lymph node metastases. With regard to whole-body assessment, we compared ^18^F-Choline-PET/CT, CeCT, and ^18^F-Fluoride-PET/CT in terms of their ability to detect bone metastases. Two cases of skeletal metastases are illustrated in Figures 4 and 5. The data regarding sensitivity are summarized in Table 4.

## 5. Correlation between ADC Values and SUV-max

With regard to local recurrences, we found a high and statistically significant inverse correlation between ADC value (7.4 × 10^−4^ ± 2 × 10^−4^ mm^2^/s, median 9.2 × 10^−4^ [7.4 × 10^−4^–1.32 × 10^−3^]) and SUV-max (3.3 ± 1.3, median 3.9 [1.4–4.8]) (*r* = −0.83, *P* = 0.04). With regard to lymph node recurrences, we found a moderate and statistically significant inverse correlation between ADC value (1.4 × 10^−3^ ± 1.1 × 10^−3^ mm^2^/s, median 9.8 × 10^−3^ [1.2 × 10^−4^–4.1 × 10^−3^]) and SUV-max (7 ± 4.1, median 6.4 [1.6–14]) (*r* = −0.6, *P* = 0.02).

## 6. Discussion

In this pilot study, we used different morphological and functional imaging modalities (i.e., mMRI, CeCT, ^18^F-Choline-PET/CT, ^18^F-Fluoride-PET/CT, and fused ^18^F-Choline-PET/MRI) to prospectively examine a small, homogeneous cohort of 21 PC patients with biochemical recurrence after first-line EBRT. The clinical implementation of ^18^F-Choline-PET/CT and mMRI in assessing recurrent PC after EBRT is currently yielding encouraging results [7, 16], and this growing evidence is confirmed by the data of our study showing high DRs for both modalities (i.e., 76% and 81%, resp.). Multimodal fusion imaging between ^18^F-Choline-PET/CT and mMRI (fused ^18^F-Choline-PET/MRI) yielded an even better DR (i.e., 86%), thus underscoring that multimodal coregistration, synchronized navigation, and combined interpretation are more valuable than the individual, separate assessment of different diagnostic techniques. Only preliminary studies [11, 12] are available on the simultaneous acquisition of PET and MRI with integrated PET/MRI scanners on PC, and, to date, only one paper has addressed the issue of multimodal fusion PET/MRI imaging (with the two modalities acquired at separate times with different scanners) in the assessment of 17 patients with primary prostate cancer [17]. To the best of our knowledge, our study was the first to investigate the diagnostic value of this hybrid technique in the assessment of PC recurrence after EBRT. Park et al. [17] performed an intermodality MRI/^11^C-Choline-PET fusion process assisted by high-resolution* ex vivo* MRI of the prostate specimen, which also offered the integration of registered histologic information. They created a parameter represented by the simple quotient of SUV over ADC value in a volume of interest within the prostate parenchyma, finding that the SUV/ADC ratio significantly increased the lesion-to-benign background contrast for tumours with Gleason grades ≥3 + 4. In our paper, we adopted an easier and more feasible coregistration technique, assisted by the new software Quanta Prostate (Camelot Biomedical Systems s.r.l., Genoa, Italy), to integrate morphological, functional, and metabolic information from different imaging modalities. With regard to the lesion-based analysis, ^18^F-Choline-PET/MRI showed significantly higher accuracy, sensitivity, and NPV than mMRI (*P* < 0.05) and CeCT (*P* < 0.05). Specifically, ^18^F-Choline-PET/MRI detected more lymph node metastases than mMRI and CeCT. This result may be due to the fact that only morphological and size criteria were adopted for defining a metastatic lymph node (i.e., short axis diameter >10 mm for an oval lymph node and diameter >8 mm for a round lymph node) on both mMRI and CeCT. In a previous pilot study [13] on 14 PC patients, Beer et al. compared ^11^C-Choline-PET SUV with ADC values of pelvic lymph nodes suspected for metastatic involvement. They found a moderate but significant inverse correlation between SUV and ADC values and a significant difference in mean ADC and SUV values between benign and malignant lymph nodes. Our results confirmed this significant inverse correlation between SUV and ADC values. However, a major limitation of DWI in PC is the lack of specificity of the ADC value in discriminating between benign and malignant lymph nodes. We did not find any significant difference in terms of diagnostic performance between ^18^F-Choline-PET/MRI and ^18^F-Choline-PET/CT, but this latter technique was able to identify only 4/6 cases of local relapse versus 6/6 with the former technique. Although the diagnostic value of T2w imaging is hampered by radiation-induced fibrosis and shrinkage of the prostate, functional MRI techniques (i.e., DWI sequences and DCE-MRI) are effective in the detection of local PC recurrence. When multimodal fusion imaging is used, perfect matching between a focus of ^18^F-Choline uptake and a suspicious mMRI finding on fused PET/MRI images may improve the diagnostic confidence of each separate diagnostic modality. Despite the low number of patients included, a significant inverse correlation was also observed between ADC and SUV values in the case of local recurrent disease. This supports the relationship in PC between the high cellularity expressed by ADC and the metabolic activity of phospholipid turnover expressed by SUV. With regard to the detection of skeletal metastases in pelvic bones, fused ^18^F-Choline-PET/MRI performed significantly better than CeCT (Table 4). In addition, on comparing CeCT, ^18^F-Choline-PET/CT, and ^18^F-Fluoride-PET/CT in the detection of bone metastases throughout the entire skeleton, we found that the highest sensitivity was obtained with ^18^F-Fluoride-PET/CT. However, a significant difference emerged only between ^18^F-Fluoride-PET/CT and CeCT. ^18^F-Choline-PET/CT proved to be slightly less sensitive than ^18^F-Fluoride-PET/CT, as previously reported [18], in which it failed to detect one small sclerotic rib metastasis.

Despite our encouraging results, some limitations should be noted. As the number of patients in our study was small, no specific sample size calculation was performed. In addition, some questions may arise with regard to the reference standard that we adopted. Indeed, no really independent tool for classifying lymph node and bone lesions was available, and, for logistic and ethical reasons, histological confirmation of metastases was not obtained in the majority of cases. However, as in two previous studies [10, 16], clinical, laboratory, and follow-up data were collectively considered and used as a standard of reference. This type of multidisciplinary follow-up is generally accepted to confirm lymph node and bone metastases. Moreover, histological confirmation was available in 3/5 patients showing only local recurrence and in 1/6 patients affected by only lymph node metastases.

## 7. Conclusions

According to our preliminary results, ^18^F-Choline-PET/MRI fusion imaging may be considered a feasible and promising diagnostic tool for detecting PC recurrence in patients showing biochemical relapse after first-line treatment with EBRT.

## Figures and Tables

**Table 1:** Characteristics of patients.

	Mean ± SD	95% CI for the mean	Median	95% for the median
Age (years)	77.2 ± 5.1	74.8 to 79	78 (70–85)	3.5 to 7.7
Mean serum PSA (ng/mL) on inclusion	5.8 ± 3.4	54.3 to 7.4	4.9 (2.2–13.4)	73.1 to 80
Gleason sum	8.1 ± 0.85	7.7 to 8.5	8 (7–9)	7 to 8
Time interval EBRT-inclusion	23.9 ± 13.4	17.8 to 30.1	24 (6–68)	17.1 to 28

**Table 2:** Patient-based analysis. DR was calculated for each single diagnostic modality in each site of disease.

Site of disease (number of patients considered)	mMRI	^ 18^F-Choline-PET/MRI	CeCT	^ 18^F-Choline-PET/CT	^ 18^F-Fluoride-PET/MRI
Local (21)	6/21 (28%)	6/21 (28%)	1/21 (5%)	4/21 (19%)	—
Lymph nodes (21)	5/21 (24%)	6/21 (28%)	5/21 (24%)	6/21 (28%)	—
Bones of the pelvis (21)	6/21 (28%)	6/21 (28%)	3/21 (14%)	6/21 (28%)	6/21 (28%)
Cumulative DR (21)	17/21 (81%)	18/21 (86%)	9/21 (43%)	16/21 (76%)	6/21 (28%)
Bone (9)	—	—	4/9 (44%)	8/9 (89%)	9/9 (100%)

**Table 3:** Lesion-based analysis and sensitivity in lesion detection according to different sites of recurrence/metastases (i.e., local recurrence, lymph nodes, and bone) obtained by mMRI, ^18^F-Choline PET/MRI, CeCT, and ^18^F-Choline PET/CT.

	mMRI	^ 18^F-Choline-PET/MRI	CeCT	^ 18^F-Choline-PET/CT
Prostate gland				
Sensitivity	6/6 (100%)	6/6 (100%)	1/6 (17%)	4/6 (67%)
Specificity	14/15 (93%)	15/15 (100%)	15/15 (100%)	15/15 (100%)
Accuracy	20/21 (95%)	21/21 (100%)	16/21 (76%)	19/21 (90%)
Lymph nodes				
Sensitivity	27/40 (67%)	40/40 (100%)	23/40 (57%)	40/40 (100%)
Specificity	15/15 (100%)	15/15 (100%)	15/15 (100%)	15/15 (100%)
Accuracy	42/55 (76%)	55/55 (100%)	38/55 (69%)	55/55 (100%)
Bone				
Sensitivity	14/14 (100%)	14/14 (100%)	6/14 (43%)	13/14 (93%)
Specificity	11/12 (92%)	11/12 (92%)	12/12 (100%)	11/12 (92%)
Accuracy	25/26 (96%)	25/26 (96%)	18/26 (69%)	25/26 (96%)

**Table 4:** Lesion-based analysis and whole-body bone metastases detection by ^18^F-Choline PET/CT, CeCT, and ^18^F-Fluoride PET/CT.

	CeCT	^ 18^F-Choline-PET/CT	*P* value
Sensitivity	15/33 (45%)	29/33 (88%)	0.0006
Specificity	24/24 (100%)	22/24 (92%)	>0.05
Accuracy	39/57 (68%)	51/57 (89%)	0.012

	^ 18^F-Choline-PET/CT	^ 18^F-Fluoride-PET/CT	*P* value

Sensitivity	29/33 (88%)	30/33 (91%)	>0.05
Specificity	22/24 (92%)	22/25 (88%)	>0.05
Accuracy	51/57 (89%)	51/57 (89%)	>0.05

	^ 18^F-Fluoride-PET/CT	CeCT	*P* value

Sensitivity	30/33 (91%)	15/33 (45%)	0.0002
Specificity	22/25 (88%)	24/24 (100%)	>0.05
Accuracy	51/57 (89%)	39/57 (68%)	0.012
