# Corrigendum to “Value of Fused ^18^F-Choline-PET/MRI to Evaluate Prostate Cancer Relapse in Patients Showing Biochemical Recurrence after EBRT: Preliminary Results”

**DOI:** 10.1155/2016/5047948

**Published:** 2016-03-20

**Authors:** Arnoldo Piccardo, Francesco Paparo, Riccardo Piccazzo, Mehrdad Naseri, Paolo Ricci, Andrea Marziano, Lorenzo Bacigalupo, Ennio Biscaldi, Gian Andrea Rollandi, Filippo Grillo-Ruggieri, Mohsen Farsad

**Affiliations:** ^1^Nuclear Medicine Department, E.O. Galliera Hospital, Mura delle Cappuccine 14, 16128 Genoa, Italy; ^2^Radiology Department, E.O. Galliera Hospital, Mura delle Cappuccine 14, 16128 Genoa, Italy; ^3^Radiotherapy Department, E.O. Galliera Hospital, Mura delle Cappuccine 14, 16128 Genoa, Italy; ^4^Nuclear Medicine Department, Azienda Sanitaria dell'Alto Adige, Via Lorenz Böhler 5, 39100 Bolzano, Italy

In the research article titled “Value of Fused ^18^F-Choline-PET/MRI to Evaluate Prostate Cancer Relapse in Patients Showing Biochemical Recurrence after EBRT: Preliminary Results” [1] the last name of the third author was wrong, because it was spelled with a “c” instead of double “c”. The correct last name is as shown above.
